# Activation of a gene network in durum wheat roots exposed to cadmium

**DOI:** 10.1186/s12870-018-1473-4

**Published:** 2018-10-16

**Authors:** Alessio Aprile, Erika Sabella, Marzia Vergine, Alessandra Genga, Maria Siciliano, Eliana Nutricati, Patrizia Rampino, Mariarosaria De Pascali, Andrea Luvisi, Antonio Miceli, Carmine Negro, Luigi De Bellis

**Affiliations:** Department of Biological and Environmental Sciences and Technologies, University of Salento, via Prov.le Monteroni 165, 73100 Lecce, Italy

**Keywords:** Cadmium, *PYE*, *ORG2*, *FIT*, Methionine salvage pathway, Nicotianamine, Durum wheat, mRNA sequencing, Heavy metals

## Abstract

**Background:**

Among cereals, durum wheat (*Triticum turgidum* L. subsp. *durum*) accumulates cadmium (Cd) at higher concentration if grown in Cd-polluted soils. Since cadmium accumulation is a risk for human health, the international trade organizations have limited the acceptable concentration of Cd in edible crops. Therefore, durum wheat cultivars accumulating low cadmium in grains should be preferred by farmers and consumers. To identify the response of durum wheat to the presence of Cd, the transcriptomes of roots and shoots of Creso and Svevo cultivars were sequenced after a 50-day exposure to 0.5 μM Cd in hydroponic solution.

**Results:**

No phytotoxic effects or biomass reduction was observed in Creso and Svevo plants at this Cd concentration. Despite this null effect, cadmium was accumulated in root tissues, in shoots and in grains suggesting a good cadmium translocation rate among tissues. The mRNA sequencing revealed a general transcriptome rearrangement after Cd treatment and more than 7000 genes were found differentially expressed in root and shoot tissues. Among these, the up-regulated genes in roots showed a clear correlation with cadmium uptake and detoxification. In particular, about three hundred genes were commonly up-regulated in Creso and Svevo roots suggesting a well defined molecular strategy characterized by the transcriptomic activation of several transcription factors mainly belonging to bHLH and WRKY families. bHLHs are probably the activators of the strong up-regulation of three *NAS* genes, responsible for the synthesis of the phytosiderophore nicotianamine (NA). Moreover, we found the overall up-regulation of the methionine salvage pathway that is tightly connected with NA synthesis and supply the S-adenosyl methionine necessary for NA biosynthesis. Finally, several vacuolar NA chelating heavy metal transporters were vigorously activated.

**Conclusions:**

In conclusion, the exposure of durum wheat to cadmium activates in roots a complex gene network involved in cadmium translocation and detoxification from heavy metals. These findings are confident with a role of nicotianamine and methionine salvage pathway in the accumulation of cadmium in durum wheat.

**Electronic supplementary material:**

The online version of this article (10.1186/s12870-018-1473-4) contains supplementary material, which is available to authorized users.

## Background

Cadmium is one of the toxic heavy metals that affect human and plants. Due to the application of phosphate fertilizers and the use of wastewaters for irrigation, the cadmium contamination of soils will increase in the future [1, 2]. Plant species could accumulate cadmium when grown on polluted soils [3]. In particular, durum wheat (*Triticum turgidum* L. subsp. *durum*) could accumulate high level of Cd in grains and, among durum wheat cultivars (cvs), a great variability was reported [4–7].

The genetic traits involved in Cd accumulation were well defined in durum wheat and a single locus (*Cdu1*) explaining 80% of the variability was identified [8, 9]. Wiebe [10] found a perfect match between the *HMA3-B1* gene (coding a P1B-ATPase transporter) and the *Cdu1* locus. Moreover, he sequenced *HMA3-B1* from high and low Cd accumulator durum wheat cvs discovering a 17 bp duplication in high-Cd genotypes causing a premature stop codon and thus, a severely truncated protein, suggesting *HMA3* as the best candidate gene for *Cdu1* locus. The HMA3 transporter alone, however, cannot explain the whole retention of cadmium in roots. For instance, the formation of stable cadmium complexes in the cytosol and vacuole should be necessary. Macfie et al. [11] evaluated the role of phytochelatins in five durum wheat near-isogenic lines, concluding that they have no role explaining a different Cd accumulation in roots.

The transcriptomic approach has revealed that plants deeply modify their transcriptomes in response to Cd stress [12–16] and these studies also indicated that transcriptional regulation of Cd tolerance-related genes is sometimes a conserved strategy among species. Previous studies have characterized several transcription factors (TFs) involved in Cd response [17] belonging to ERF, bZIP, WRKY, and bHLH TF families, supporting the complexity of plants response to Cd stress.

With the aim to identify the basal conserved molecular mechanisms that regulate cadmium uptake in durum wheat, we used two cvs (Creso and Svevo). The transcriptomes of roots and shoots of Creso and Svevo cvs, cultivated in the absence and in the presence of Cd, were compared and their commonly modulated genes were discussed since they identify the basal conserved molecular response to Cd in durum wheat.

## Methods

### Genetic materials

To identify common molecular strategies in durum wheat (*Triticum turgidum* L. subsp. *durum*) in response to Cd, the transcriptomes of two durum wheat cultivars (Creso and Svevo) were analyzed.

Creso and Svevo accession numbers are, respectively, K-53049 and RICP-01C0107074, and all pedigree information is browsable at CYMMIT database (http://www.wheatpedigree.net). The two cvs were selected because of their wide utilization and for their different behavior in Cd accumulation in shoot tissues and grains. In particular, Svevo demonstrated to accumulate higher level of Cd in grains and leaves than Creso. On the contrary, Creso accumulates more Cd in roots than Svevo [6, 7].

### Experimental design

For a rigorous Cd administration to Creso and Svevo cvs, a hydroponic system was set up. After surface sterilization, seeds were germinated in Petri dishes with moist filter paper, in the dark at 8 °C. After germination (6–7 days), seedlings were placed in plastic pots (10 × 10 × 50 cm) filled with perlite, moistened with deionized water, and immediately transferred to the hydroponic system as described by Harris and Taylor [18]. Each pot contained three seedlings and for each treatment, three different pots were considered (three biological replicates). The positions of the pots in the growth chamber were completely randomized.

The hydroponic solution was given at regular intervals (4 h), irrigating for 5 min. In this way, the perlite substrate was constantly moistened with hydroponic solution, but stagnation was avoided. Plants were grown in two separate Fitotron® Growth Rooms (Weiss Technik, U.K.) under controlled conditions [7] (see Additional file 1 for a detailed description).

The nutrient solution was prepared using reverse osmosis (RO) water (< 30 μS cm^ − 1^) and contained 1.0 mM Ca(NO_3_)_2_, 0.3 mM Mg(NO_3_)_2_, 0.3 mM NH_4_NO_3_, 0.25 mM KNO_3_, 0.1 mM K_2_HPO_4_, 0.1 mM K_2_SO_4_, 50 μM KCl, 100 μM Fe(NO_3_)_3_, 10 μM H_3_BO_3_, 0.2 μM Na_2_MoO_4_, 10 μM ZnSO_4_, 2 μM CuSO_4_, 1 μM MnSO_4_, 0.1 μM NiCl_2_, 138.6 μM N- (2-hydroxyethyl) ethylenediaminetriacetic acid (HEDTA), 1.42 mM KOH, and 2 mM 2-(N-morpholino) ethanesulfonic acid (MES); the pH of the nutrient solution was constantly maintained between 5.6 and 5.9. HEDTA was added to the nutrient solution to reproduce the environmental availability of free metals [19]. Plants were treated with 0.5 μM CdCl_2_, whereas control plants were cultivated without Cd in hydroponic solution. As described by Harris and Taylor [18] this Cd treatment exposes the roots to a not toxic Cd concentration. Hydroponic solution was constantly aerated.

One plant for each pot (three for each treatment) was sampled 50 days after germination, at tillering stage (roots and shoots). Roots were easily extracted from perlite substrate and washed manually to remove the perlite beads adherent to roots. Grains (from two plants/pot) were collected at maturity. Samples were washed in RO water for 30 s after harvesting. Samples for mRNA sequencing were frozen in liquid nitrogen and then harvested at − 80 °C.

### Biomass analysis

Roots and shoots were dried at 100 °C to a constant weight, after which dry weight was determined. The yield was measured at maturity by electronic weight scale (*n* = 3).

### Cd concentration determination

Cd concentration was determined as described by Vergine and coworkers [7]. Samples were dried and 0.1 g was digested in a solution containing 6 mL of trace-metal-grade concentrated HNO_3_ and 1 mL of 30% (*v*/*v*) H_2_O_2_, in a microwave digestion system Milestone MLS 1200 MEGA (FKV, Sorisole, BG, Italy). Following Massadeh and Snook [20], 10 mL of deionized water was added after cooling, and the solution was filtered through a Whatman filter paper 40 into a 25 mL volumetric flask. The volume obtained was topped up to the mark with deionized water. Cd was determined by graphite furnace atomic absorption spectroscopy (GF-AAS, PinAAcle, PerkinElmer, USA). Quantitative analysis was achieved by interpolating the relevant calibration curves prepared from aqueous solutions of metal standards in the same acid concentration to minimize matrix effects. The method detection limit (MDL) was 0.08 μg L^− 1^. Concentrations of the species were obtained with the removal of the average level present in blank samples. The calculated concentration of a specific species was quantified if it was larger than the standard deviation σB of the blank; otherwise, a threshold value equal to σB was considered. In cases in which the concentration was below the MDL or not detectable above the average variability of the field blanks, a concentration value equal to the maximum between the MDL and σB was assumed.

### mRNA sequencing and data mining

Total RNA was extracted from root and shoot tissues using TRIZOL reagent according to the method published by Aprile et al. [21]. To assess RNA quality and quantity, several dilutions of each sample were analyzed using the Agilent RNA 6000 nano Kit and Agilent Bioanalyzer 2100.

RNA sequencing and bioinformatic analyses were performed by IGA Technology Services (Udine, Italy). Three biological replicates of roots and shoots of Creso and Svevo grown in hydroponic conditions without and with Cd (0.5 μM) were sequenced for each condition, resulting in 24 samples. Sequencing was performed in 100 bp single-end mode on HiSeq2500 (Illumina, San Diego, CA). Subsequently, alignments were performed with TopHat2 [22, 23] on *Triticum* reference genome/transcriptome owned by IGA Technology Services, using default parameters. Raw RNA-sequencing data were deposited in the public database “ArrayExpress”, at European Bioinformatics Institute (EMBL-EBI) (https://www.ebi.ac.uk/arrayexpress). The accession code is E-MTAB-7266.

Median alignment rate was 85.7%. Homology-based functional annotation of *Triticum* genes was performed using BLAST [24] on *Arabidopsis thaliana* genome, setting the E-value threshold at ≤10^− 5^. Gene expression, i.e. the relative abundances of transcripts was estimated by Cufflinks [23]. Pair-wise differential expression analysis was performed by Cuffdiff [25]. Differentially expressed contigs were identified through a Welch t-test with Benjamini and Hochberg false discovery rate correction for multiple tests. A contig was considered differentially expressed (DEG) if it satisfied the following conditions: q-value (FDR-adjusted *p*-value) < 0.001, two-fold change (FC) in FPKM (Fragments Per Kilobase Million) value, and an FPKM value of at least 5 in at least one samples. Applying a filtering method on FPKM > 5.0, we found that 49,536 genes are expressed in at least one condition.

RNA-sequencing data were validated by qRT-PCR analysis. Ten random contig sequences were selected for comparison between RNA-seq data and qPCR. qRT-PCR reactions were performed with SYBR Green fluorescence detection in a qPCR thermal cycler (ABI PRISM 7900HT, Applied Biosystems). Each reaction was prepared using 5 μL from a 0.2 ng/μL dilution of cDNA derived from the RT reaction, 12.5 μL of SYBR Green PCR Master Mix (Applied Biosystems), 1 μM forward and reverse primers, in a total volume of 25 μL. The cycling conditions were: 10 min at 95 °C, followed by 40 cycles of 95 °C for 15 s and 60 °C for 1 min with the final dissociation at 95 °C for 15 s, 60 °C for 30 s and 95 °C for 15 s.

The contig8902 annotated as a gene coding for a voltage-dependent anion channel 1 was selected based on the lowest coefficient of variation (CV = standard deviation/mean) observed, and used as reference gene in qRT-PCR.

RNA-seq data mining was performed using g:Profiler [26], a public web server for characterizing and manipulating gene lists resulting from high-throughput genomic data. g:Profiler allowed to identify the statistical enrichment of functional categories among the lists of up- and down-regulated genes, setting a *p*-value< 0.001.

STRING [27] is a biological database and web resource that allows to identify the known and predicted protein-protein interactions. The version 10.0 contains information on about 9.6 million proteins from more than 2000 organisms. Lists of up-regulated genes in durum wheat roots were analyzed with STRING to find the protein interactions and to shed light on possible regulatory networks.

All data sets for this study are included in the manuscript and in the Additional files 1, 2, 3, 4, 5, 6, 7 and 8.

## Results

**Fig. 1:**
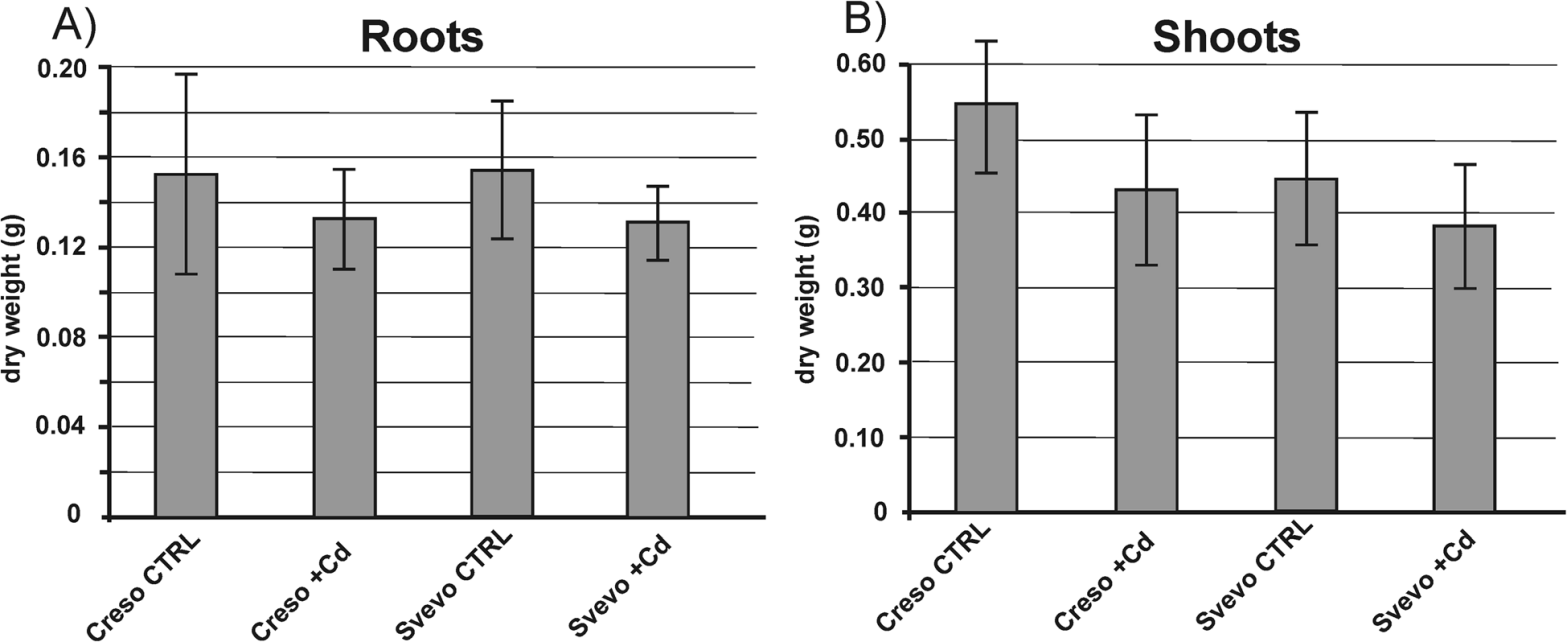
Root and shoot dry weight biomass of Creso and Svevo. Root (**a**) and shoot (**b**) dry weight biomass of Creso and Svevo durum wheat plants grown in standard hydroponic solution and in the presence of Cd 0.5 μM. Samples were collected 50 days after germination (at the beginning of tillering stage). Statistical analysis did not show any statistical difference (*p*-value< 0.05, *n* = 3)

Samples were collected 50 days after germination, at the beginning of tillering stage, from plants grown in hydroponic solutions with sub-lethal concentrations of Cd (0.5 μM). Analysis of variance (2-way ANOVA, *p*-value< 0.05, *n* = 3) did not highlight significant changes in growth between treated plants and control plants for roots (Fig. 1a), as well as for shoot tissues (Fig. 1b).

A similar behavior was observed by Harris and Taylor [28] who described the growth of two isogenic lines (accumulating opposite quantity of cadmium in grains) in terms of biomass, pointing out that there are no significant differences between the two plants if cultivated in the presence or in absence of cadmium.

**Fig. 2:**
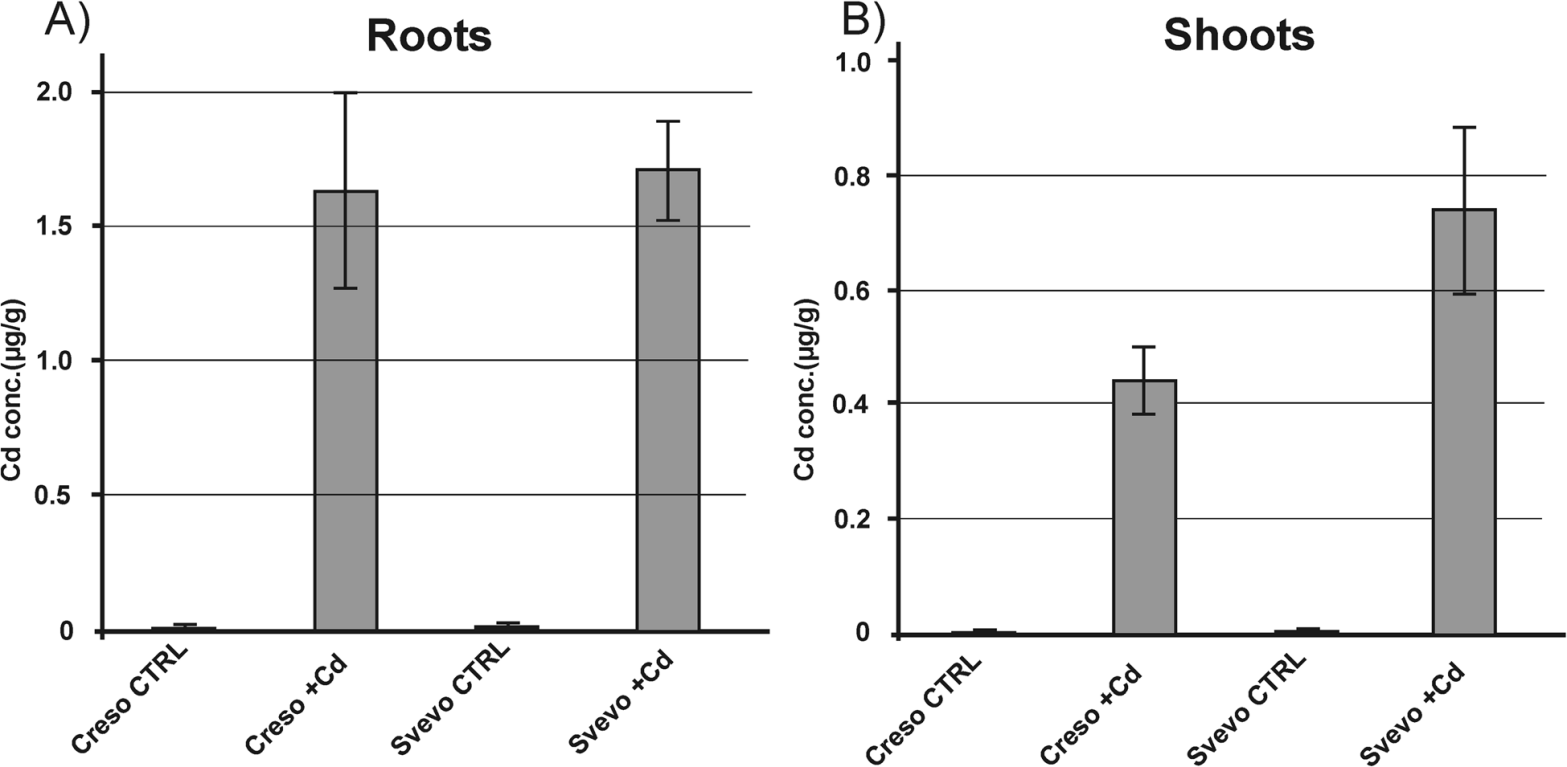
Cd concentration in roots and shoots of Creso and Svevo cvs. Cadmium concentrations of durum wheat genotypes grown in standard hydroponic solution in the presence of 0.5 μM Cd. Roots (**a**) and shoots (**b**) were collected 50 days after germination (at the beginning of tillering stage). Cd concentration was quantified by GF-AAS. Statistical analysis was performed through ANOVA (*p*-value< 0.05, *n* = 3)

The same samples were analyzed by atomic absorption (GF-AAS) to quantify the level of accumulated Cd in roots and shoots: as expected, Cd concentration is higher in roots than in shoots (Fig. 2). This phenomenon has been already described by Harris and Taylor [18]. Moreover, Vergine et al. [7] and Arduini et al. [6], reported no significant differences in roots between Creso and Svevo. Also in our experiment, Creso and Svevo genotypes did not show significant differences in Cd accumulation in roots (1.62 and 1.69 μg/g dry weight) (Fig. 2a). On the contrary, there were differences in shoot Cd concentrations; samples of Creso showed a lower Cd accumulation compared to Svevo samples: 0.43 μg of Cd/g dry weight in Creso, 0.73 μg/g in Svevo (Fig. 2b).

**Fig. 3:**
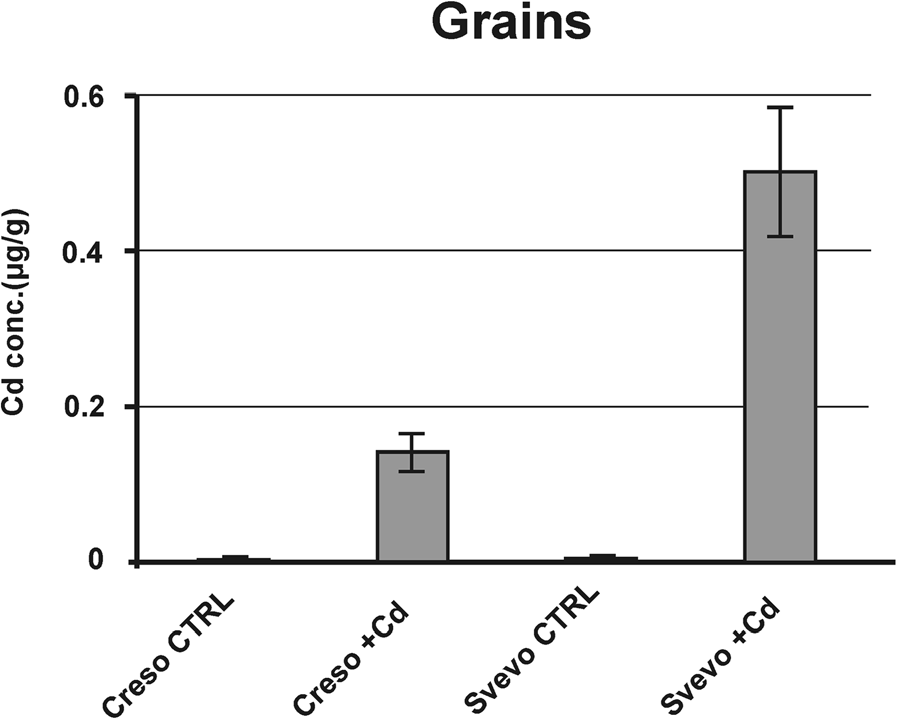
Cd concentration in grains of Creso and Svevo cvs. Cd concentration in grains of Creso and Svevo durum wheat grown in standard hydroponic solution and in the presence of 0.5 μM Cd. Statistical analysis was performed through ANOVA (*p*-value< 0.05, *n* = 3)

The content of Cd was also analyzed in mature grains (Fig. 3). Creso accumulated low levels of Cd (0.16 ± 0.02 μg/g) while Svevo accumulated 0.50 ± 0.12 μg/g. These data fit well with those reported by Arduini and coworkers (2014) on kernels harvested at maturity.

### RNA-sequencing and quality of data

The Illumina HiSeq2500 generated 717.4 single-end reads with a median 28.3 million (M) of reads/sample (min 21.2 M, max 52.9 M), reduced to 716.0 M single-end high-quality reads after the trimming with ERNE [29] and removal of adapter sequences with Cutadapt [30]. Transcript abundance for each gene was calculated as Fragments Per Kilobase Million (FPKM) (Additional file 2 and Additional file 3). The Pearson correlation coefficients were calculated between pairs of biological replicates within each condition. The values ranged from 0.955 to 0.999 suggesting a high level of correlation among biological replicates.

RNA-seq validation was carried out on eight random selected contigs. Although the magnitude of the transcript expression was, to some extent, different between RNA-sequencing and qRT-PCR, all tested genes showed the same expression trend with the two methods. The Pearson product-moment correlation coefficients between RNA-sequencing and qRT-PCR data was 0.885, confirming the good similarity between the two methods.

**Fig. 4:**
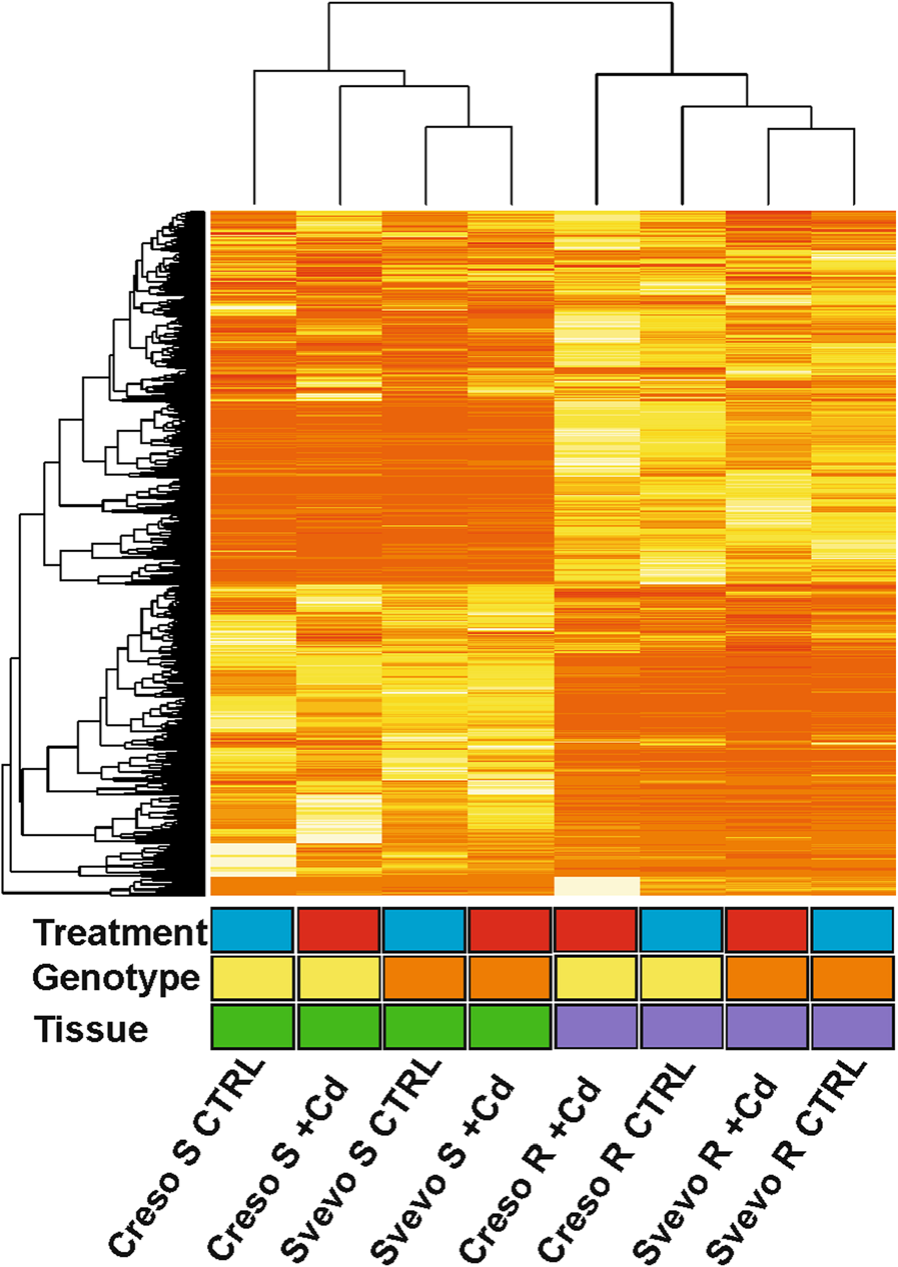
Unsupervised clustering of dataset of Creso and Svevo transcriptomes. Heatmap relative to the mRNA-seq dataset relative to Creso and Svevo. Two main branches grouped samples from the same tissue: shoots (S, green boxes) and roots (R, purple boxes). Among each branch the samples grouped following the genotypes: Creso (yellow boxes) and Svevo (orange boxes). Treatment (CTRL and + Cd = 0.5 μM) was the experiment variable that caused the lower variance among samples. Blue boxes = CTRL; red boxes = Cd-treated samples

To evaluate data quality and the effect of experiment variables (treatment, genotype, and tissue) on the data set, an unsupervised clustering analysis was done and the relative heatmap was generated (Fig. 4). Clustered data suggest that the main source of variation is the tissue type since samples from same tissue were grouped together and clustered in two main clades (shoot on the left and roots on the right). The second cluster level is between genotypes, and samples of Creso genotype were grouped together as well as Svevo genotype samples. Treatment is the condition that produced a lower variance among samples.

### Transcriptome changing in plants treated with cadmium

To identify the molecular mechanisms underlying the translocation/detoxification of Cd in wheat, the transcriptomes of roots and shoots of the two wheat cvs Creso and Svevo, cultivated in the absence and in the presence of cadmium were compared.

In particular, four comparisons were done with the aim to find common/conserved molecular strategies:

i) CTRL vs Cd-treated samples of Creso (root tissue). This comparison allows to identify modulated genes after Cd treatment in the roots.
  The comparison identified 1626 differentially expressed genes (DEG), among them 1203 were up-regulated and 423 were down-regulated by Cd treatment.
ii) CTRL vs Cd-treated samples of Svevo (root tissue). This comparison allows to identify modulated genes after Cd treatment in the roots.
  The comparison identified 2549 DEG, among them 2188 were up-regulated and 361 were down-regulated by Cd treatment.

Taken together, data from Creso and Svevo roots highlighted the modulation of 3807 genes in response to Cd exposure. In particular, durum wheat root responses to cadmium are regulated mainly by up-regulation events, since about 80% of modulated genes were up-regulated in Creso and Svevo cvs.

**Fig. 5:**
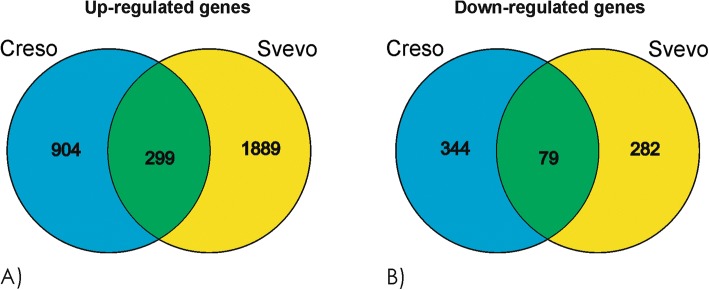
Venn diagram of (**a**) up- and (**b**) down-regulated genes in roots of Creso and Svevo cvs

The overlapping genes between the lists obtained from i) and ii) comparisons were represented as Venn diagrams (Fig. 5a and b) to point out the conserved response between the two genotypes. Respectively, 299 and 79 genes were up-regulated or down-regulated in the same way in the two genotypes.

iii) CTRL vs Cd-treated samples of Creso (shoot tissue). This comparison allows to identify modulated genes after Cd treatment in the shoots of cv Creso: a total of 1351 were modulated by Cd treatment, 540 of them were up-regulated and 811 were down-regulated.
iv) CTRL vs Cd-treated samples of Svevo (shoot tissue). This comparison allows to identify modulated genes after Cd treatment in the shoots of cv Svevo. The comparison highlighted 3235 differentially expressed genes, 1072 were up-regulated and 2163 were down-regulated.

**Fig. 6:**
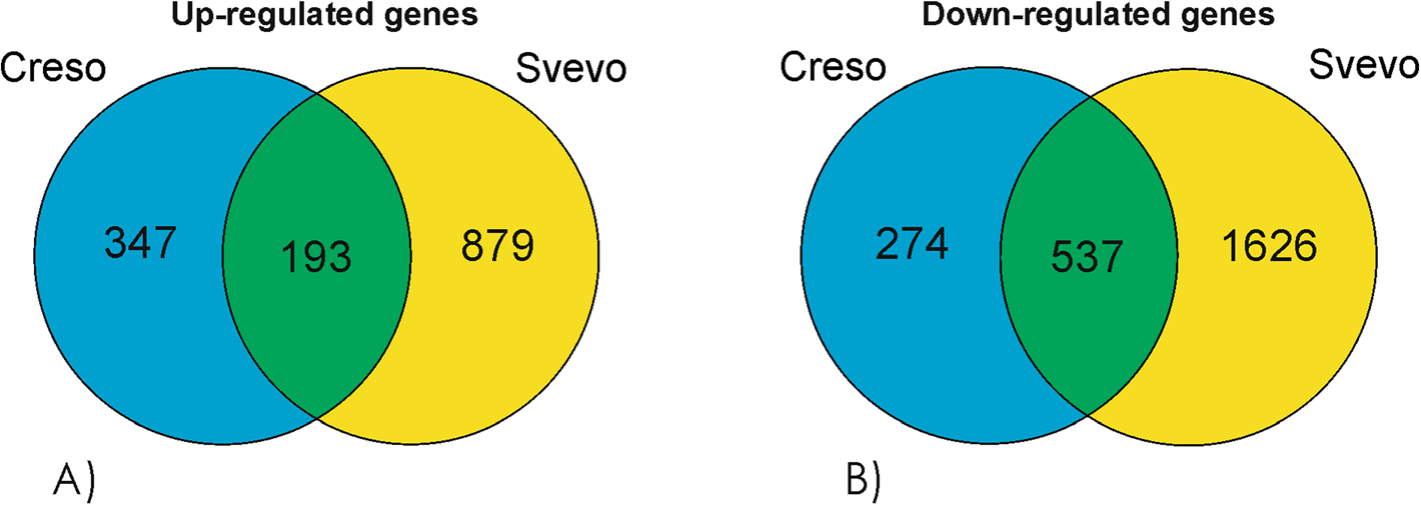
Venn diagram of (**a**) up- and (**b**) down-regulated genes in shoots of Creso and Svevo cvs

The Venn diagram representation (Fig. 6a and b) shows that Creso and Svevo share only 14% (193 genes) of the total up-regulated genes (1419 genes). On the contrary, among the down-regulated genes (a total of 2437), 22% (537 genes) is commonly modulated by Creso and Svevo genotypes.

### Data mining and identification of molecular mechanisms in response to cadmium exposure

The analysis of the transcriptomes of Creso and Svevo in response to Cd treatment identified about 7100 differentially expressed genes in root and shoot tissues. To identify the conserved molecular mechanism involved in detoxification or translocation of Cd in durum wheat, we focused our attention on the modulated genes that were up- or down-regulated in both genotypes. As reported in Figs. 5 and 6, we obtained four lists (Additional files 4, 5, 6 and 7) of commonly regulated genes: the up-regulated and down-regulated genes in roots (299 and 79 genes, respectively), and the up-regulated and down-regulated genes in shoots (193 and 537 genes, respectively).

To better understand the underlying biological processes for each commonly regulated gene, a statistical functional enrichment analysis (g:Profiler, [26]) was carried out.

### Commonly up-regulated genes in roots

The gene set enrichment analysis carried out on the commonly up-regulated genes in roots, highlighted 9 enriched categories (*p*-value < 0.001) (Additional file 8). As expected, some categories involved in metal ion response, transport and homeostasis were gene enriched such as “*response to inorganic substance*”, “*inorganic cation transmembrane transport”, “transition metal ion transport”, “cellular response to iron ion starvation” and “transition metal ion homeostasis”.* These categories grouped 34 different genes and most of them were annotated as transporters (Table 1) and transcription factors (Table 2).

**Table 1:** List of up-regulated contigs in Creso and Svevo wheat roots with high sequence similarity to membrane transporters of heavy metals

Contig ID	Creso roots		FC	Svevo roots		FC	TAIR AGI CODE	TAIR Annotation
	CTRL	+Cd		CTRL	+Cd			
contig45935	5.2	13.7	2.6	3.9	8.7	2.3	AT1G10970	zinc transporter 4 precursor (ZIP4)
contig48191	2.2	6.6	3.0	2.0	5.1	2.6	AT1G10970	
contig73320	1.5	5.9	3.9	1.1	8.7	7.9	AT1G10970	
contig16227	7.2	29.5	4.1	6.3	13.9	2.2	AT1G47240	NRAMP metal ion transporter 2 (NRAMP2)
contig30944	2.6	15.0	5.8	2.4	7.2	3.0	AT1G51340	MATE efflux family protein (MATE)
contig13545	18.2	42.7	2.3	12.9	40.5	3.1	AT1G63440	heavy metal atpase 5 (HMA5)
contig17301	5.8	15.1	2.6	5.2	11.6	2.2	AT1G63440	
contig20319	13.9	47.7	3.4	9.8	44.8	4.6	AT1G63440	
contig40560	17.1	47.9	2.8	14.4	37.5	2.6	AT1G63440	
contig5883	14.1	33.8	2.4	12.0	24.5	2.0	AT1G63440	
contig59715	1.3	10.2	7.7	2.0	24.5	12.1	AT1G76800	vacuolar iron transporter (VIT)
contig6853	5.3	33.9	6.4	7.5	47.0	6.2	AT1G76800	
contig25958	7.0	25.1	3.6	5.7	12.6	2.2	AT3G20870	ZIP metal ion transporter family (ZTP29)
contig61554	24.7	111.8	4.5	17.1	80.3	4.7	AT3G20870	
contig6172	11.8	48.7	4.1	8.4	26.0	3.1	AT3G20870	
contig36800	2.0	70.0	35.5	1.3	28.3	22.5	AT3G43790	zinc induced facilitator-like 2 (ZIFL2)
contig38345	11.4	124.9	10.9	6.4	49.2	7.6	AT3G43790	
contig54958	3.3	40.8	12.5	1.8	16.5	9.0	AT3G43790	
contig143856	5.1	23.5	4.6	2.8	6.8	2.4	AT4G24120	yellow stripe like 1 (YSL1)
contig36593	2.4	13.4	5.6	2.5	14.1	5.8	AT5G03570	iron regulated 2 (IREG2)
contig47439	5.7	57.4	10.1	3.0	40.8	13.5	AT5G03570	
contig155	3.1	121.9	39.4	1.0	34.5	33.0	AT5G13740	zinc induced facilitator 1 (ZIF1)
contig15565	3.1	30.1	9.8	2.1	10.8	5.1	AT5G13750	zinc induced facilitator-like 1 (ZIFL1)
contig75088	9.0	166.7	18.5	3.3	77.3	23.3	AT5G13750	
contig81708	1.3	14.4	10.9	1.3	5.2	4.0	AT5G13750	
contig31191	5.3	10.7	2.0	4.3	12.9	3.0	AT5G20650	copper transporter 5 (COPT5)
contig64300	3.2	16.6	5.1	2.7	5.5	2.0	AT5G23760	copper transport protein family (HPP7)
contig25370	29.7	113.5	3.8	26.3	65.5	2.5	AT5G24380	yellow stripe like 2 (YSL2)
contig26249	17.6	80.0	4.5	7.7	42.6	5.5	AT5G39040	ABC transporter B type member 27 (ABCB27)

In “CTRL” and “+Cd” columns the expression values (FPKM) are reported. In “FC” columns the relative fold changes are reported. The last two columns report the AGI code and the annotation of the most similar Arabidopsis gene to the wheat contig

**Table 2:** List of up-regulated contigs in Creso and Svevo wheat roots with high sequence similarity to transcription factors

Contig ID	Creso roots		FC	Svevo roots		FC	TAIR AGI CODE	TAIR Annotation
	CTRL	+Cd		CTRL	+Cd			
contig33526	1.7	2.8	1.7	2.8	5.7	2.0	AT1G27730	salt tolerance zinc finger (ZAT10)
contig41708	5.7	13.5	2.3	8.3	19.9	2.4	AT1G27730	
contig57171	4.1	9.1	2.2	7.1	20.2	2.8	AT1G27730	
contig72518	0.9	2.0	2.3	1.5	6.8	4.4	AT1G27730	
contig12928	16.3	40.6	2.5	13.1	41.1	3.1	AT2G28160	basic helix-loop-helix (bHLH) DNA-binding (bHLH29/FIT)
contig50497	9.1	15.6	1.7	6.9	21.3	3.1	AT2G28160	
contig69766	6.1	12.6	2.1	5.6	15.6	2.8	AT2G28160	
contig33459	6.3	15.3	2.4	8.1	25.1	3.1	AT2G38470	WRKY DNA-binding protein 33 (WRKY33)
contig42013	6.7	16.9	2.5	10.2	30.0	2.9	AT2G38470	
contig50500	4.6	10.2	2.2	5.9	13.4	2.3	AT2G38470	
contig54713	1.4	1.7	1.3	1.4	16.5	11.5	AT2G38470	
contig64297	3.2	9.3	2.9	6.1	13.1	2.1	AT3G20310	ethylene response factor 7 (ERF7)
contig81176	1.1	3.5	3.3	2.2	10.0	4.5	AT3G20310	
contig25001	4.6	16.3	3.5	5.5	11.1	2.0	AT3G47640	basic helix-loop-helix (bHLH) DNA-binding (bHLH47/PYE)
contig49051	0.7	1.4	2.1	1.6	7.0	4.4	AT3G47640	
contig119028	0.9	7.8	8.9	0.7	4.7	6.6	AT3G56970	basic helix-loop-helix (bHLH) DNA-binding (bHLH38/ORG2)
contig59732	4.7	16.0	3.4	4.6	15.5	3.4	AT3G56970	
contig45646	3.6	8.1	2.2	5.3	12.0	2.3	AT4G23810	WRKY DNA-binding protein 53 (WRKY53)
contig25913	4.8	11.9	2.5	7.6	17.4	2.3	AT5G24110	WRKY DNA-binding protein 30 (WRKY30)
contig64256	3.5	8.9	2.5	5.2	11.2	2.2	AT5G24110	
contig67994	2.0	6.8	3.4	5.0	10.8	2.2	AT5G24110	
contig74889	1.7	3.6	2.1	2.7	5.1	1.9	AT5G24110	

As reported in Table 1, several kinds of transporters were up-regulated in durum wheat roots in response to cadmium: there are copper transporters (AT5G23760 Copper transport family – HPP7, AT5G20650 Copper transporter 5 - COPT5, AT1G63440 Copper-transporting ATPase - HMA5, AT1G66240 – Homolog of anti-oxidant 1 – ATX1), iron transporters (AT5G03570- Iron regulated 2 – IREG2, AT1G76800- Vacuolar iron transporter homolog - VIT), several zinc transporters (AT5G13750- Zinc transporter- ZIFL1, AT5G13740- Zinc Induced Facilitator 1 - ZIF1, AT3G43790- Zinc induced facilitator-like 2 - ZIFL2, AT3G20870- Zinc transporter ZTP29, AT1G10970- Zinc transporter 4 – ZIP4), aluminum transporters (AT5G39040- ABC transporter B family member 27 – ABCB27, AT1G51340- MATE efflux family protein) and manganese transporters (AT1G47240- Metal transporter NRAMP2). Two particular kinds of transporters were also up-regulated by cadmium in durum wheat roots namely Yellow Stripe-Like protein YSL1 (AT4G24120) and YSL2 (AT5G24380). They are localized to vacuole membranes and are able to transport metal-nicotianamine (NA) complexes [31, 32].

This strong up-regulation of membrane heavy metal transporters suggests that the cadmium exposure activates many non-specific different molecular mechanisms involved in the translocation or compartmentalization of heavy metals.

Eight transcription factors belonging to the same previously indicated enriched functional categories were also identified among up-regulated genes. In particular we found transcriptional factors belonging to WRKY-family (AT5G24110 – WRKY30, AT4G23810 – WRKY53 and AT2G38470 – WRKY33), basic/helix-loop-helix (bHLH) (AT3G56970 – bHLH38/ORG2, AT3G47640 – bHLH47/PYE and AT2G28160 - bHLH29/FIT), one belongs to ERF family (AT3G20310 – ERF7) as well as one transcriptional repressor involved in abiotic stress response (AT1G27730 – ZAT10). Table 2 reports the expression levels and the relative fold change of each contig related to the eight transcription factors.

Moreover, among the 299 commonly up-regulated genes in roots of Creso and Svevo cvs, we noticed other transcription factors that could act in response to cadmium exposure since they belong to the same families above described: ERF9 (AT5G44210), ERF11 (AT1G28370), WRKY18 and WRKY41.

On the basis on the gene set enrichment analysis, we focused our attention on other four functional categories that resulted over-represented: “*nicotianamine metabolic process*”, “*methionine metabolic process*”, “*amino acid salvage*” and “*aromatic amino acid family catabolic process*”.

The category “*nicotianamine metabolic process*” is a very small category since it has only five entries in the Arabidopsis GO database, nevertheless in this experiment three genes belonging to this functional category were up-regulated by cadmium in roots. These genes code all for nicotianamine synthase proteins (AT1G56080 – NAS2, AT1G09240 – NAS3, AT1G56430 – NAS4) and RNA sequencing allowed to find 17 contigs with high similarity to the sequences of these three *NAS* genes. After cadmium treatment, the expression level of *NAS2*, *NAS3,* and *NAS4* rose up dramatically and the average FC observed is about 60, reaching expression levels close to 800 FPKM (contig46013) (Table 3).

**Table 3:** List of up-regulated contigs in wheat roots with high sequence similarity to Arabidopsis nicotianamine synthase genes

Contig ID	Creso roots		FC	Svevo roots		FC	TAIR AGI CODE	TAIR Annotation
	CTRL	+Cd		CTRL	+Cd			
contig18293	7.6	274.5	36.1	1.5	146.9	99.7	AT5G56080	nicotianamine synthase 2 (NAS2)
contig18375	40.0	414.7	10.4	8.9	291.7	32.7	AT5G56080	
contig18424	10.1	415.2	41.2	3.0	591.7	198.1	AT1G09240	nicotianamine synthase 3 (NAS3)
contig40805	19.3	363.4	18.8	5.2	322.2	62.4	AT1G09240	
contig5670	1.9	41.2	21.4	0.3	28.9	85.4	AT1G09240	
contig6866	7.5	266.9	35.8	2.3	190.1	82.8	AT1G09240	
contig11228	2.9	10.8	3.7	2.2	8.8	3.9	AT1G56430	nicotianamine synthase 4 (NAS4)
contig13016	4.1	155.3	38.2	0.6	101.3	177.4	AT1G56430	
contig18348	0.4	31.4	73.3	2.8	191.8	67.5	AT1G56430	
contig27606	12.6	151.0	12.0	2.5	120.7	48.6	AT1G56430	
contig46013	27.1	771.3	28.5	8.0	691.1	86.6	AT1G56430	
contig51008	5.1	174.3	34.2	1.4	128.5	91.9	AT1G56430	
contig5669	9.4	210.9	22.4	1.8	257.5	141.5	AT1G56430	
contig56695	2.1	57.5	27.5	0.6	47.8	82.0	AT1G56430	
contig62096	7.9	251.7	31.9	2.0	340.6	173.0	AT1G56430	
contig6432	9.3	353.4	38.1	2.3	208.9	89.0	AT1G56430	
contig76098	12.1	182.6	15.0	2.6	45.0	17.6	AT1G56430	

The three remaining enriched categories (“*methionine metabolic process*”, “*amino acid salvage*” and “*aromatic amino acid family catabolic process*”) grouped 11 genes that belong mainly to the same biosynthetic pathway, the methionine salvage cycle.

The methionine salvage cycle consists of 9 enzymatic steps and, since the amount of methionine is typically limiting in cells and de novo synthesis of methionine is energetically expensive, it is important to recycle this amino acid. In this way, the methionine regeneration from MTA plays an important role in sustaining the continued production of the siderophore nicotianamine. The genes coding for NAS (Table 3), MTN, MTI, MTK, DEP, ARD, TAT1 and TAT2 were all up-regulated in roots (Table 4).

**Table 4:** List of up-regulated contigs in wheat roots with high sequence similarity to genes involved in methionine metabolic process

Contig ID	Creso roots		FC	Svevo roots		FC	TAIR AGI CODE	TAIR Annotation
	CTRL	+Cd		CTRL	+Cd			
contig2986	230.7	717.3	3.1	256.7	648.1	2.5	AT4G14716	Acireductone dioxygenase 1 (ARD1)
contig930	260.6	895.9	3.4	265.2	878.6	3.3	AT4G14716	
contig39444	2.0	15.0	7.6	3.4	8.0	2.3	AT1G50110	Branched-Chain Aminotransferase 6 (BCAT6)
contig13983	4.0	40.5	10.2	4.9	14.1	2.9	AT3G25900	Homocysteine S-methyltransferase (HMT-1)
contig25432	1.6	18.0	11.2	1.6	10.5	6.7	AT1G80360	Methionine aminotransferase (ISS1)
contig10788	13.9	62.8	4.5	15.4	44.3	2.9	AT2G05830	5-methylthioribose kinase 1 (MTI1)
contig10789	25.4	61.0	2.4	23.8	53.0	2.2	AT2G05830	
contig32510	3.9	23.0	5.8	3.2	10.3	3.3	AT1G49820	S-methyl-5-thioribose kinase (MTK)
contig5064	26.8	106.2	4.0	26.3	64.2	2.4	AT1G49820	
contig80693	1.9	7.1	3.8	0.9	17.6	19.4	AT1G49820	
contig11517	53.5	139.1	2.6	54.4	161.3	3.0	AT4G34840	Methylthioadenosine nucleosidase 2 (MTN2)
contig11518	37.2	108.5	2.9	33.9	101.6	3.0	AT4G34840	
contig14879	2.6	8.0	3.1	4.7	11.6	2.5	AT3G53260	Phenylalanine ammonia-lyase 2 (PAL2)
contig104004	2.3	12.7	5.6	1.1	5.5	5.0	AT5G53970	Tyrosine transaminase 1 (TAT1)
contig16069	30.5	102.5	3.4	16.9	49.8	2.9	AT5G53970	
contig20039	32.8	481.2	14.7	8.1	272.7	33.8	AT5G53970	
contig3346	14.6	60.0	4.1	10.0	30.5	3.1	AT5G53970	
contig7573	10.4	209.0	20.1	2.2	121.7	56.5	AT5G53970	
contig33842	4.1	11.4	2.8	3.1	7.1	2.3	AT5G36160	Tyrosine transaminase 2 (TAT2)
contig1980	64.6	129.0	2.0	61.8	125.9	2.0	AT5G53850	dehydratase / enolase / phosphatase (DEP)

### Commonly down-regulated genes in roots

Creso and Svevo cvs shared 79 commonly down-regulated contigs, corresponding to 53 different genes (Additional file 5). As well as for up-regulated genes, a functional enrichment analysis was carried out, but no functional category was found enriched (*p*-value< 0.001).

### Commonly up-regulated genes in shoots

In leaves, the transcriptome comparison between Creso and Svevo revealed 193 commonly up-regulated genes between the two cultivars (Additional file 6). Anyway, in such gene list, we did not find any over-represented functional category (*p*-value< 0.001).

### Commonly down-regulated genes in shoots

Creso and Svevo shoots have a large number of commonly down-regulated genes (537, see Additional file 7). The genes of this list fall into categories related to the plant signaling (“*response to stimulus*” GO:0050896, “*signal transduction*” GO:0007165, “*protein phosphorylation*” GO:0006468 and “*cell communication*” GO:0007154) and carbohydrate pathway (“*cellular carbohydrate metabolic process*” GO:0044262). These categories indicated a general transcriptome reaction to an abiotic stress but did not identify a specific defense strategy against cadmium.

### STRING analysis

**Fig. 7:**
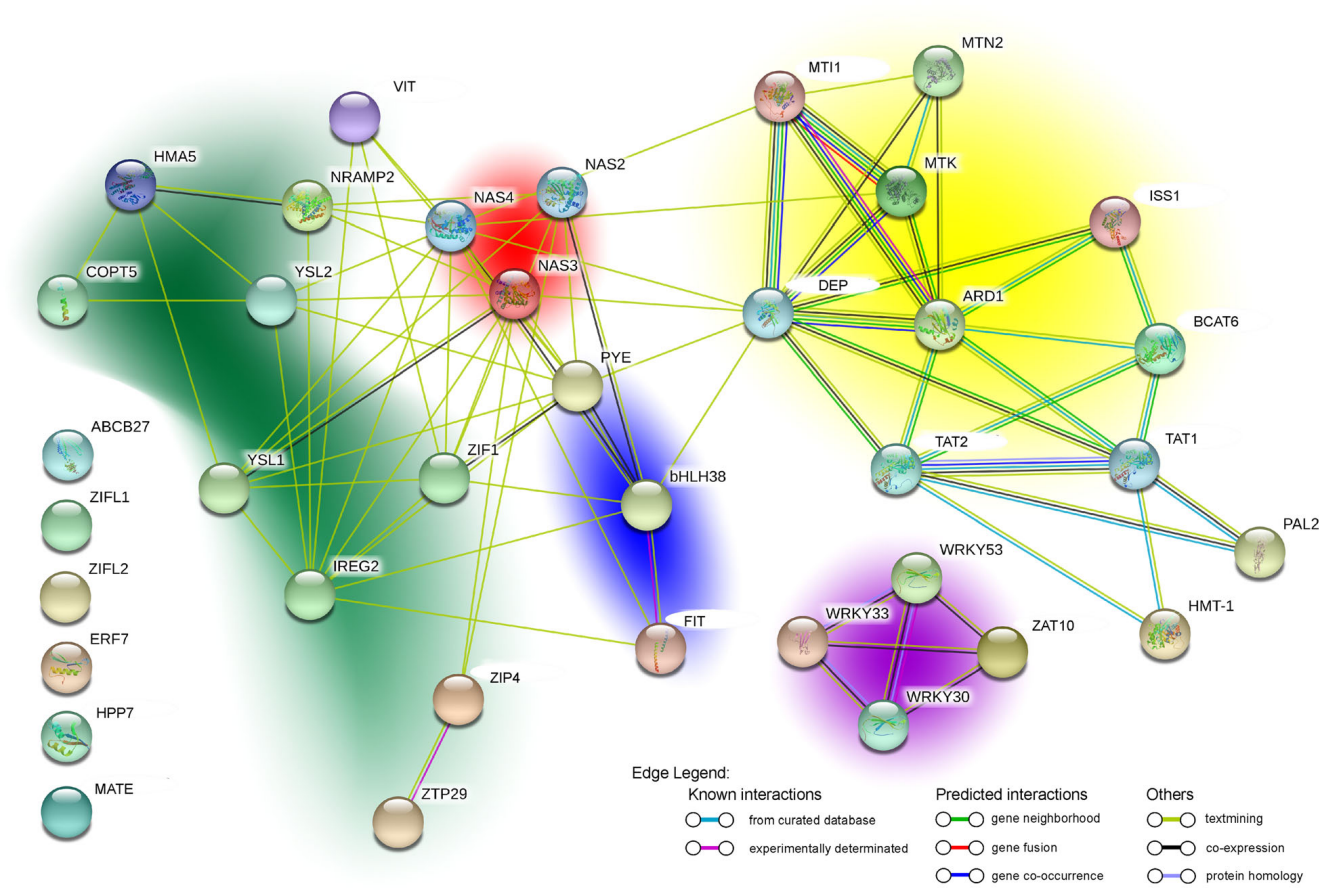
Protein network based on the cadmium up-regulated genes in durum wheat roots. Green cloud indicates heavy metals transporters; red cloud groups the three NAS; blue cloud groups the three bHLH family transcription factors; yellow cloud clusters components of the methionine salvage pathway; purple cloud groups together the WRKY transcription factors

A new bioinformatic tool called STRING allows to easily get information about protein-protein interactions [27]. This database finds protein interactions at multiple levels such as (i) known experimental interactions, (ii) pathway knowledge, (iii) automated text-mining, (iv) de novo prediction by a number of algorithms using genomic information, (v) and by co-expression analysis. In order to obtain a graphical interpretation of the numerous interactions among the proteins coded by the genes characterized in this study, a STRING analysis was done and the results are presented in Fig. 7. The bHLH transcription factors are localized at the center of the network, as well as the three NAS, highlighting the high number of connections with the other proteins. The groups of bHLHs are strongly connected to NAS and both are linked to the methionine salvage pathway as well as to heavy metal transporters, suggesting their key role in the transcriptional regulation/connection.

## Discussion

In general, the data reported in this work indicate that despite the null effect of the used Cd concentration on toxicity and biomass, Cd is actually accumulated in Creso and Svevo in root tissues (about 1.65 μg/g dry weight for both genotypes), in shoots (0.43 and 0.73 μg/g dry weight, respectively) and in grains (0.16 and 0.50 μg/g dry weight, respectively), suggesting a good cadmium translocation rate among tissues, without any particular physiological effect on plant growth. In particular, the cv Svevo showed a high concentration in grains, exceeding the maximum concentration (0.2 μg of Cd/g dry weight) allowed by the Codex Alimentarius Commission [33]. In fact, some durum wheat cultivars, even if grown in soils with low or moderate Cd contamination, may accumulate high, and sometimes over the limit, levels of Cd in grains [5, 7].

The best strategy to avoid Cd grain contamination is to unravel the molecular mechanisms involved in Cd accumulation and, consequently, to develop breeding strategies or genetic modifications to obtain cultivars that do not translocate Cd up to the grains [34]. The genetic explanation for the high-low Cd phenotype in durum wheat was well studied. Genetically, Cd accumulation in grains is a process controlled by many genes that could have a combined effect on uptake, sequestration, detoxification and translocation. Anyway, in durum wheat, Cd accumulation is regulated by the major locus *Cdu1*, which is localized on 5BL chromosome [9, 10] and unfolds 80% of the high-low Cd phenotype in grain [8]. The gene responsible for the *Cdu1* locus is not yet described, although it could code for a tonoplast transporter in root cells. Wiebe [10] found a complete linkage between the gene *HMA3-B1* (coding a P_1_B-ATPase) and the *Cdu-B1* locus. Moreover, he sequenced the *HMA3-B1* from high and low Cd durum wheat accumulators discovering a 17 bp duplication in high-Cd genotypes causing a pre-mature stop codon and thus, a severely truncated protein. Thus suggesting *HMA3* as the best candidate gene for *Cdu1* locus. With the aim to evaluate the behavior of this gene in roots and shoots of Creso and Svevo cvs, we performed a BLAST analysis of the *HMA3* sequence against Creso and Svevo sequenced transcriptomes, finding a contig (contig37808, Additional file 1) with high sequence similarity to *HMA3-B1*. Anyway, we did not find any difference in transcription regulation of this contig since we observed a generally low level of expression (near to the background) in roots and no expression at all in leaves. These data do not support a central role of *HMA3-B1* in cadmium accumulation. However, since we collected mRNA 50 days after germination, the *HMA3* gene could be regulated during a different stadium of the durum wheat life cycle or it could be subjected to post-transcriptional regulation. In fact, also Macfie and colleagues [11] hypothesized that a single transporter as HMA3 cannot explain the retention of cadmium in the roots. Moreover, the plant would also require the formation of stable cadmium complexes in the cytosol and vacuole [11].

Our data on mRNA pools of roots and shoots of the two durum wheat cvs grown with and without cadmium revealed a general common transcriptome re-organization, that could be a good explanation of how durum wheat could detoxify cells from cadmium. In this experiment, we employed two commercial durum wheat cultivars that do not share a common pedigree or common ancestors and, for this reason, they were not suitable to find genetic differences between the two genotypes. In fact, many differences in gene expression, not related to Cd tolerance/accumulation, might also be expected when two commercial cultivars are transcriptionally compared.

In roots, the response to cadmium is mainly regulated by up-regulation events (Fig. 5a and b). On the contrary, in leaves, the cadmium exposure induces down-regulation of about 2400 genes, while the up-regulated genes are “only” 1400 (Fig. 6a and b). Moreover, the analysis of functional categories on these genes indicate a strong cadmium response correlation between the up-regulated genes in roots and the molecular mechanisms that are at the basis of the cadmium translocation and detoxification, all these observations suggest peculiar molecular responses involving the activation of specific transcription factors, transporters, phytosiderophores and activation of pathways sustaining the synthesis of phytosiderophores.

The STRING analysis highlighted a complex gene network among these genes where transcription factors and NAS genes are in the middle of the network.

### Transcription factors

Transcriptomic changes analysis allowed to identify various transcription factors (TFs) involved in Cd plant response [17] belonging to several TF families (ERF, bZIP, WRKY, bHLH), supporting the complexity of plants response of plants to Cd stress. Accordingly, after Cd exposure, we found many differentially expressed TFs (Table 2). Anyway, the up-regulation levels are not very strong: the highest FPKM reported was 41.1, and it is relative to the *bHLH29* (contig12928) gene, whereas the observed fold changes were generally not higher than 3.0.

In Arabidopsis, *ERF* (Ethylene Responsive Factor) genes were identified in response to cadmium [35] and in *Phaseolus vulgaris,* the *PvERF15* has a key role in the up-regulation of cadmium response genes [16]. Among the up-regulated genes in roots of Creso and Svevo, we found four different contigs related to three ERF transcription factors (ERF7, ERF9, and ERF11) suggesting a possible role in activation of downstream cadmium response genes. In particular in radish, *ERF7* is co-regulated with *YSL1*, a metal-nicotianamine (NA) transporter for metal ion transport [36].

The members of the WRKY transcription factors family were also described to be involved in the response to cadmium and ethylene production [17]. Cadmium treatment induced the biosynthesis of ACC and ethylene in *Arabidopsis thaliana* plants mainly via the increased expression of *ACS2* and *ACS6* [37] and, due to the direct binding of WRKY33 to the W-boxes in the promoters of these two genes, WRKY33 is directly involved in the activation of *ACS2* and *ACS6* [38]. The data here reported indicate that in durum wheat, *WRKY33* (AT2G38470) was up-regulated in roots by cadmium exposure (Table 2), although ACC synthase genes are not differentially expressed. Within WRKY transcription factor family, *WRKY18*, *WRKY30*, *WRKY41,* and *WRKY53* were up-regulated by cadmium in roots. As reported by other authors WRKY 18 and WRKY41, regulate the H_2_S signaling pathway in plants, allowing them to cope with cadmium stress [39]. Our data support that the WRKY family has a key role in regulating transcriptional response to cadmium stress also in durum wheat.

The transcription factors named C(2)H(2)-zinc finger are involved in modulating the defense response of plants to abiotic stress. The over-expression of *ZAT10*, for example, induces production of reactive oxygen species and enhances the tolerance of plants to abiotic stress [40]. Another member of zinc-finger family, *ZAT6*, activates phytochelatins synthesis-related genes and positively regulate Cd accumulation [41]. Also in durum wheat, we identified two contigs with high sequence similarity to *ZAT10* sequence (AT1G27730) (Table 2) that were up-regulated by cadmium.

The basic helix-loop-helix (bHLH) transcription factors are regulatory components in gene expression networks and are involved in a wide variety of processes such as responses to abiotic stress [42]. For instance, *At*bHLH29/FIT interacts with *At*bHLH38/ORG2 to enhance the Cd tolerance in Arabidopsis, decreasing cadmium transport from roots to shoots and improving the iron homeostasis and concentration of shoots as reported by Wu et al. [43]. The same authors also reported that co-overexpression of *FIT* and *ORG2* constitutively activated the expression of *Heavy Metal Associated3* (HMA3) and *Iron Regulated Gene2* (*IREG2*), which are involved in the heavy metal detoxification in Arabidopsis. Moreover, co-overexpression of *FIT* and *ORG2* enhanced the expression of *nicotianamine synthase 1* (*NAS1*) and *NAS2*, resulting in the accumulation of nicotiananamine, a crucial chelator for Fe transportation and homeostasis [44]. Another transcription factor belonging to bHLH family, *AtbHLH47* (named *POPEYE/PYE*) is also involved in iron and heavy metals homeostasis [44].

In roots of Creso and Svevo cvs we found that *FIT*, *ORG2*, and *PYE* (Table 2) were all up-regulated by cadmium, as well as the downstream genes *HMA5*, *IREG2, NAS2*, *NAS3* and *NAS4* (Table 1 and Table 3) suggesting that the molecular mechanisms regulated by bHLH transcription factors are well conserved between Arabidopsis and durum wheat.

Taken together all the mRNA sequencing data on transcription factors in roots of durum wheat reveal a complex regulation network where the bHLH, WRKY and ERF families could play key roles in the transcriptional activation of phytochelatins, transporters, and heavy metal transporters.

### Heavy metals transporters

Table 1 lists the heavy metals transporters that were up-regulated by cadmium exposure in durum wheat roots, the data reported highlight how cadmium treatment is able to activate the expression of many, not-specific, different transporters. In fact, we found that expression levels of copper, iron, zinc, aluminum, and manganese transporters were up-regulated by Cd treatment. Moreover, the nicotianamine transporter genes (*YSL1* and *YSL2*) and the NA vacuolar transporter genes (*ZIF* and *ZIF-like* genes) were up-regulated, too. In Arabidopsis *ZIF1*, coding for a transporter is transcriptionally regulated by the transcription factors *PYE* [44]. In durum wheat, according to our data, this gene exhibiting the higher level of up-regulation among transporters genes, is co-regulated with *PYE*, suggesting that the co-regulation between *PYE* and* ZIF* is conserved among plant species.

### Nicotianamine synthase genes and methionine salvage pathway

In Arabidopsis FIT, ORG2 and PYE were described as regulators of nicotianamine synthase 1 and 2 (*NAS1*, *NAS2*) transcription [43]. The mRNA sequencing data of Creso and Svevo cvs revealed an astonishing up-regulation of three NAS genes (*NAS2*, *NAS3,* and *NAS4*)*.* For all of them, after cadmium treatment, the level of expression rose up to 500 FPKM and up-regulation levels higher than 100-times (Table 3) between control and treated samples were observed. In Arabidopsis, *NAS4* is required for Cd resistance [45] and was found constitutively up-regulated in the cadmium hyper-accumulator *Arabidopsis halleri* [35].

Nicotianamine synthase enzymes catalyze the synthesis of nicotianamine (NA) by the trimerization of S-adenosyl-L-methionine (SAM) [46]. NA is a non-proteinogenic amino acid with a high binding affinity for a range of transition metal cations and is the precursor of mugineic acid family phytosiderophores in cereals [47]. Moreover, NA is a phytosiderophore transported through cell membranes and vacuole by YSL and ZIF1 transporters [48]; in the two durum wheat cvs studied *YSL1*, *YSL2*, *ZIF1*, *ZIF-like1,* and *ZIF-like2* are also up-regulated as mentioned above (Table 1).

**Fig. 8:**
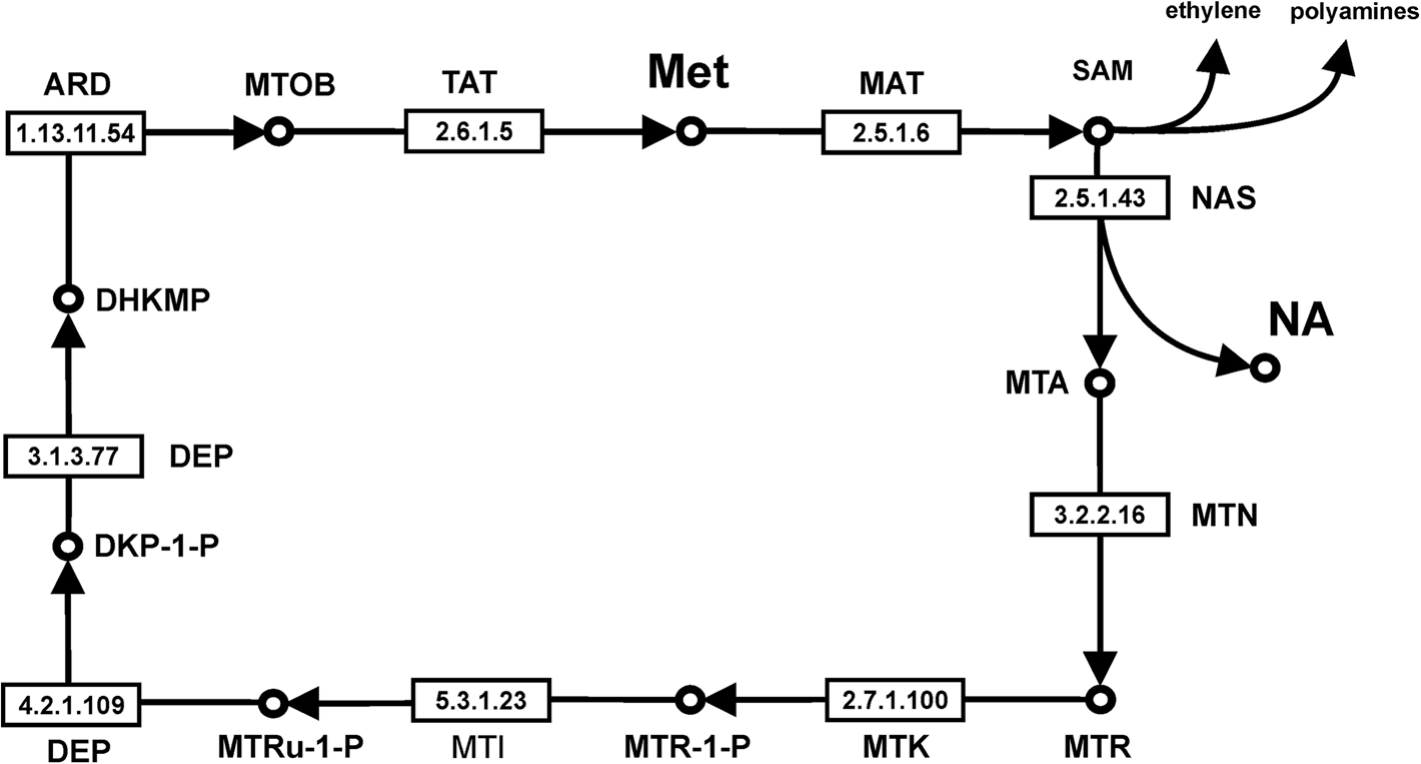
Methionine Salvage Pathway. Main reactions and their links of the methionine salvage pathway (inferred from KEGG database, [51]). Numbers in the boxes refer to enzyme IUBMB nomenclature. Met: Methionine; SAM: S-adenosyl methionine, NA: nicotianamine; MTA: 5′-methylthioadenosine; MTR: S-methyl-thio-D-ribose; MTR-1-P: S-methyl-thio-D-ribose 1-phosphate; MTRu-1-P: S-methyl-thio-D-ribulose 1-phosphate; DKP-1-P: 2,3-Diketo-5-methylthiopentyl-1-phosphate; DHKMP: 1,2-Dihydroxy-5-methyl-thiopentene;MTOB: 4-methylthio-2-oxobutanoate; MAT: S-adenosyl-L-methionine synthase; NAS: nicotianamine synthase; MTN: methylthioadenosine nucleosidase; MTK: MTR kinase; MTI: 5-methylthioribose-1-phosphate isomerase; DEP: dehydratase/enolase/phosphatase; ARD: acireductone dioxygenase; TAT: tyrosine aminiotransferase

During the synthesis of each NA, three methionines are consumed, producing three S-adenosylmethionine (AdoMet) releasing three toxic by-products molecules of 5′-methylthioadenosine (MTA) [49]. Since the amount of methionine is typically limiting in cells and a new synthesis is energetically expensive, this amino acid is recycled by cells [50] through the methionine salvage pathway (MSP) (Fig. 8).

In eukaryotic cells, MSP takes place in the cytoplasm and is characterized by nine enzymatic steps, catalyzed by eight enzymes. Starting from methionine, the first step is catalyzed by S-adenosyl-L-methionine synthase (MAT, EC 2.5.1.6) catalyzing formation of SAM from methionine and ATP. Our mRNA sequencing findings identified six different contigs with high sequence similarity to Arabidopsis *MAT* (AT3G17390). Their level of expression was very high in roots (ranging between 100 and 700 FPKM) and lower in leaves (25–150 FPKM), but these contigs were not differentially expressed between control and cadmium-treated samples. SAM is the substrate for many pathways such as the biosynthesis of ethylene, polyamines, and NA. We found that *NAS* (EC 2.5.1.43) genes responsible for the synthesis of the phytosiderophore NA and MTA are strongly up-regulated. MTA is recycled by MTN (methylthioadenosine nucleosidase, EC 3.2.2.16) producing S-methyl-thio-D-ribose (MTR) and adenine. We sequenced the mRNA of three different *MTN*-related (AT4G34840) genes and two of them resulted to be up-regulated in roots of Creso and Svevo (Table 4 and Additional file 2). MTR is phosphorylated by MTK (MTR kinase, EC 2.7.1.100) and converted in MTR-1P; durum wheat roots mRNA sequencing identified three cadmium up-regulated contigs of *MTK*-related genes. MTR-1P is then converted in MTRu-1-P by 5-methylthioribose-1-phosphate isomerase (MTI, EC 5.3.1.23), We found two contigs with high sequence similarity to an Arabidopsis *MTI* (AT2G05830) and both were up-regulated. The sixth and seventh steps of this cycle are catalyzed by one enzyme with a trifunctional dehydratase/enolase/phosphatase activity, called DEP (EC 4.2.1.109 and EC 3.1.3.77). This enzyme converts MTRu-1-P in DHKMP (1,2 dihydroxy-3-keto-5-methyl-thiopentene). We found several contigs with high sequence similarity to Arabidopsis *DEP *(AT5G53850), and two of them were up-regulated in roots of Creso and Svevo cvs. The next step converts DHKMP in MTOB (4-methylthio-2-oxobutanoate) and is catalyzed by the enzyme acireductone dioxygenase (ARD, EC 1.13.11.54). ARD is a metalloenzyme and requires iron to convert DHKMP to MTOB, but if ARD is coupled with Ni, it catalyzes an off-pathway reaction whose products are methylthio-propionate, carbon monoxide, and formate [52]. In Arabidopsis, this enzyme is coded by the *ARD1* (AT4G14716) gene and we found two contigs *ARD1*-related with a high level of expression (230–900 FPKM) in roots, up-regulated by cadmium (Table 4). Tyrosine aminotransferase (TAT, EC 2.6.1.5) catalyzes the final step of the methionine salvage pathway [53]. In Creso and Svevo transcriptome several contigs with high sequence similarity to Arabidopsis *TAT1* and *TAT2* were sequenced. In particular contigs relative to *TAT1* showed a strong up-regulation in cadmium-treated roots.

In conclusion, the massive up-regulation of genes of the methionine salvage pathway, the vigorous induction of NAS genes and the up-regulation of vacuolar NA transporters suggest the hypothesis that the phytosiderophore NA has a central role in Cd response at durum wheat root level.

## Conclusions

The cadmium exposure in durum wheat activates a complex gene network at root level.

mRNA sequencing of transcriptomes of Creso and Svevo durum wheat cultivars revealed four main responses that are all well connected each other:

1. Slight activation of several transcription factors mainly belonging to bHLH and WRKY families;
2. Strong up-regulation of three *NAS* genes that probably sustain the production of the phytosiderophore NA;
3. Strong up-regulation of the methionine salvage pathway that is tightly connected with NA synthesis and supply the SAM necessary for NA biosynthesis;
4. Overall up-regulation of different heavy metal transporters; in particular, the NA vacuolar transporters *ZIF1*, *ZIF-like* genes, and *YSL2* were vigorously activated.

The mRNA sequencing on roots and shoots of durum wheat reveals conserved response to cadmium at the root level and such response is probably regulated by bHLH transcription factors that could induce the production of the phytosiderophore NA able to chelate heavy metals. The cadmium-NA chelates could then enter into the vacuoles through the ZIF and YSL transporters.

Moreover, wheat roots respond to Cd exposure by increasing the sulfur recovery pathway, mainly by regulation of genes involved in the methionine salvage pathway. This transcriptional regulation could be an adaptive response required to ensure a sufficient supply of sulfur compounds during Cd-induced nicotianamine production.

This molecular machine is probably activated to avoid cadmium translocation from roots to upper tissues through cadmium compartmentalization into the root vacuoles.

To validate this hypothesis and the central role of nicotianamine as cadmium chelator, further experiments are needed to quantify nicotianamine after Cd treatment and to demonstrate its vacuole co-localization with cadmium.

## Additional files

Additional file 1: Growing conditions used in Fitotron® Growth Rooms (Weiss Technik, UK). * = dark conditions. In this *.docx file are reported the growing conditions used to cultivate Creso and Svevo plants. (DOCX 17 kb)

Additional file 2: Level of expression (FPKM) of root samples of Creso and Svevo cvs durum. In this *.xlsx file are reported the expression levels of about 180,000 contigs in roots of the Creso and Svevo cvs. The first column enlists the contigs identification numbers. From column B to E are reported the mean expression values (FPKM) observed in the different genotypes and treatments. (XLSX 9887 kb)

Additional file 3: Level of expression (FPKM) of shoot samples of Creso and Svevo cvs. In this *.xlsx file are reported the expression levels of about 180,000 contigs in leaves of the Creso and Svevo cvs. The first column enlists the contigs identification numbers. From column B to E are reported the mean expression values (FPKM) observed in the different genotypes and treatments. (XLSX 9732 kb)

Additional file 4: List of the 299 commonly up-regulated genes in roots of Creso and Svevo cvs. In this *.xlsx file are reported the expression levels of the 299 contigs that are commonly up-regulated by cadmium treatment in roots of the Creso and Svevo cvs. The first column enlists the contigs identification numbers. From column B to I are reported the mean expression values (FPKM) observed in the different genotypes, tissues and treatments. From column J to M are reported the relative annotation of each contig based on GO and TAIR databases. (XLSX 50 kb)

Additional file 5: List of the 79 commonly down-regulated genes in roots of Creso and Svevo cvs. In this *.xlsx file are reported the expression levels of the 79 contigs that are commonly down-regulated by cadmium treatment in roots of the Creso and Svevo cvs. The first column enlists the contigs identification numbers. From column B to I are reported the mean expression values (FPKM) observed in the different genotypes, tissues and treatments. From column J to M are reported the relative annotation of each contig based on GO and TAIR databases. (XLSX 22 kb)

Additional file 6: List of the 193 commonly up-regulated genes in shoots of Creso and Svevo cvs. In this *.xlsx file are reported the expression levels of the 193 contigs that are commonly up-regulated by cadmium treatment in shoots of the Creso and Svevo cvs. The first column enlists the contigs identification numbers. From column B to I are reported the mean expression values (FPKM) observed in the different genotypes, tissues and treatments. From column J to M are reported the relative annotation of each contig based on GO and TAIR databases. (XLSX 39 kb)

Additional file 7: List of the 540 commonly down-regulated genes in shoots of Creso and Svevo cvs. In this *.xlsx file are reported the expression levels of the 540 contigs that are commonly down-regulated by cadmium treatment in shoots of the Creso and Svevo cvs. The first column enlists the contigs identification numbers. From column B to I are reported the mean expression values (FPKM) observed in the different genotypes, tissues and treatments. From column J to M are reported the relative annotation of each contig based on GO and TAIR databases. (XLSX 90 kb)

Additional file 8: List of enriched categories among the commonly up-regulated genes in roots of Creso and Svevo cvs. In this *.xlsx file are reported the list of enriched categories obtained by g:Profiler analysis (*p*-value< 0.001). (XLSX 9 kb)

## Abbreviations

ARD: Acireductone dioxygenase
bHLH: Basic Helix-Loop-Helix
DEG: Differentially expressed genes
DEP: Dehydratase/enolase/phosphatase
DHKMP: 1,2-Dihydroxy-5-methyl-thiopentene
DKP-1-P: 2,3-Diketo-5-methylthiopentyl-1-phosphate
ERF: Ethylene responsive factor
FC: Fold Change
FPKM: Fragments Per Kilobase Million
GF-AAS: Graphite furnace atomic absorption
HMA: Heavy Metal Associated protein
MAT: S-adenosyl-L-methionine synthase
MTA: 5′-methylthioadenosine
MTI: 5-methylthioribose-1-phosphate isomerase
MTK: MTR kinase
MTN: Methylthioadenosine nucleosidase
MTOB: 4-Methylthio-2-oxobutanoate
MTR: S-Methyl-thio-D-ribose
MTR-1-P: S-methyl-thio-D-ribose 1-phosphate
MTRu-1-P: S-methyl-thio-D-ribulose 1-phosphate
NA: Nicotianamine
NAS: Nicotianamine synthase
NAS: Nicotianamine synthase
SAM: S-adenosyl methionine
TAT: Tyrosine aminiotransferase
