# Supplementary material for: Activation of a gene network in durum wheat roots exposed to cadmium
Source: BMC Plant Biol. 2018 Oct 16;18:238. doi: 10.1186/s12870-018-1473-4 (PMC6192290; doi:10.1186/s12870-018-1473-4)
Supplement: Supplementary file 1 — Growing conditions used in Fitotron® Growth Rooms (Weiss Technik, UK). * = dark conditions. In this *.docx file are reported the growing conditions used to cultivate Creso and Svevo plants. (DOCX 17 kb) [file 12870_2018_1473_MOESM1_ESM.docx]

| **Stage** | **T (°C)**  **day/night** | **RH %**  **day/night** | **Photoperiod (h)**  **day/night** | **Time (d)** | **Light intensity**  **μmol m^-2^ s^-1^** |
| --- | --- | --- | --- | --- | --- |
| ***Germination*** | 8 | 70 | * | 8-10 | - |
| ***Seedling growth*** | 10/8 | 60 | 10/14 | 25 | 300 |
| ***Tillering*** | 14/10 | 60 | 13/11 | 15 | 300 |
| ***Stem elongation*** | 15/12 | 60 | 15/9 | 15 | 600 |
| ***Booting*** | 18/15 | 50 | 16/8 | 10 | 600 |
| ***Ear emergence*** | 20/16 | 50 | 16/8 | 12 | 600 |
| ***Flowering*** | 22/18 | 50 | 16/8 | 10 | 600 |
| ***Milk development*** | 23/20 | 50 | 16/8 | 12 | 600 |
| ***Dough development*** | 25/20 | 50 | 16/8 | 21 | 900 |
| ***Ripening*** | 28/22 | 40 | 16/8 | 15 | 900 |

**Additional file 1:** Growing conditions used in Fitotron® Growth Rooms (Weiss Technik, UK). *= dark conditions.
